# A Robust Rerank Approach for Feature Selection and Its Application to Pooling-Based GWA Studies

**DOI:** 10.1155/2013/860673

**Published:** 2013-04-04

**Authors:** Jia-Rou Liu, Po-Hsiu Kuo, Hung Hung

**Affiliations:** ^1^Institute of Statistical Science, Academia Sinica, Taipei 11529, Taiwan; ^2^Institute of Epidemiology and Preventive Medicine, National Taiwan University, Taipei 10055, Taiwan; ^3^Research Center of Genes, Environment and Human Health, National Taiwan University, Taipei 10055, Taiwan

## Abstract

Large-*p*-small-*n* datasets are commonly encountered in modern biomedical studies. To detect the difference between two groups, conventional methods would fail to apply due to the instability in estimating variances in *t*-test and a high proportion of tied values in AUC (area under the receiver operating characteristic curve) estimates. The significance analysis of microarrays (SAM) may also not be satisfactory, since its performance is sensitive to the tuning parameter, and its selection is not straightforward. In this work, we propose a robust rerank approach to overcome the above-mentioned diffculties. In particular, we obtain a rank-based statistic for each feature based on the concept of “rank-over-variable.” Techniques of “random subset” and “rerank” are then iteratively applied to rank features, and the leading features will be selected for further studies. The proposed re-rank approach is especially applicable for large-*p*-small-*n* datasets. Moreover, it is insensitive to the selection of tuning parameters, which is an appealing property for practical implementation. Simulation studies and real data analysis of pooling-based genome wide association (GWA) studies demonstrate the usefulness of our method.

## 1. Introduction

Recently, many researches encounter the problem where the data objects have an extremely large number of features while the available sample size is relatively small. In view of the number of features, unless there is a large sample size, conventional statistical methods that based on asymptotic theory are not applicable. This is so-called the curse of dimensionality. To improve the accuracy and efficiency of data analysis, it is helpful to reduce the number of features before fitting statistical models. Various dimension reduction methods have been proposed, which can be categorized into two categories: feature extraction and feature selection [1]. The former is to transform a high dimensional data into a lower dimensional space, while the latter is to select a subset of the original features. For example, principle component analysis (PCA) [2] is one of the most commonly used feature extraction methods. However, the meanings of the extracted principle components are often difficult to interpret. Alternatively, feature selection methods aim to choose a subset of features which do not alter the original explanation [3, 4]. In the field of bioinformatics, statistical techniques are usually used to be a preprocessing step for the purpose of identifying relevant features with disease status. After identifying a set of susceptible genetic markers, their biological meanings or functions need to be verified by further studies. In this view, feature selection seems to be more appropriate than feature extraction due to the advantage of interpretability.

To detect the difference between two groups, one approach is fold change [5]. It is calculated simply as the ratio of the sample means of two groups. Its drawback, however, is that it ignores the variance of each group so that the statistical power is usually poor. In contrast, *t*-test takes into account the sample variances to detect mean change between two groups, and it is very powerful when the normality assumption holds. However, the data is not always normally distributed especially for the case of small sample size. The instability in estimating variances in this situation would also make type-I error inflated and fail to apply. The Significance Analysis of Microarrays (SAM) [6] is proposed to improve this drawback through adding a small positive number *s*_0_ to the denominator of the *t*-test statistic. However, it is still difficult to apply since its detection power is sensitive to *s*_0_ value and, to the best of our knowledge, there is no optimal data-driven approach to select *s*_0_.

Nonparametric methods are distribution-free and robust to the presence of outliers. To achieve robustness, these methods consider the ranks of data instead of the original measurements. Unfortunately, traditional “rank-over-sample” methods such as Wilcoxon rank sum test or area under the receiver operating characteristic curve (AUC) would fail to deal with the large-*p*-small-*n* datasets. This can be seen by considering a case-control study with *n*_1_ cases and *n*_0_ controls, where there are at most *n*_0_ *n*_1_ distinct AUC values which can be much smaller than the number of features. In this case, the high proportion of tied values would make it hard to construct a ranking list. To improve the detection power while keeping the robustness, methods based on the idea of “rank-over-variable” were proposed including Rank Product [7] and Rank Test [8]. In these methods, the original data points are still replaced by their ranks, but the rank here is defined for each feature by its position in the list of sorted variables of a single subject. As a consequence, the possible ranking values would range between one and the number of variables, which decreases the occurrence of tied values while keeping the robustness. Compared with unstably estimating variances in *t*-test, rank-over-variable methods do not involve the estimation of variance but it has been reported to be able to detect both changes in mean and correlation between two groups [8]. Motivated by the advantages of rank-over-variable, the aim of this work is to propose a robust rerank approach for feature selection. As will become clear later, the proposed rerank approach is especially applicable for large-*p*-small-*n* datasets and is not sensitive to the selection of tuning parameters. 

The rest of this paper is organized as follows. In Section 2, based on the idea of rank-over-variable, we propose a robust rerank approach to create a ranking list for feature selection. Numerical studies are conducted in Section 3 to verify that our rerank approach does outperform AUC, *t*-test, and SAM. The paper is ended with conclusions in Section 4.

## 2. Inference Procedure

### 2.1. Rerank Approach

 Consider a case-control study that examines *p* markers with *n*_1_ cases and *n*_0_ controls. Let *X*_*ij*_ and *Y*_*ij*_ be the continuous measurement for marker *j*, *j* = 1,…, *p*, of subject *i* in the case and control groups, respectively. The goal is to identify those markers which are truly associated with disease. To construct a ranking list of markers, we consider a rank-based statistic by modifying the method of Alvo et al. [8]. Define the centered markers by

(1) $$\begin{array}{c} X_{ij}^{*}=X_{ij}-\mu_{j},\;\;Y_{ij}^{*}=Y_{ij}-\mu_{j}, \end{array}$$

where

(2) $$\begin{array}{c} \mu_{j}=\frac{\sum_{i=1}^{n_{1}}X_{ij}+\sum_{i=1}^{n_{0}}Y_{ij}}{n_{1}+n_{0}} \end{array}$$

is the overall sample mean of the *j*th marker. Let *R*_X*_(*i*, *j*) be the rank of *X*_ij_* among {*X*_ij_*, *j* = 1,…, *p*} for subject *i* in decreasing order, and *R*_Y*_(*i*, *j*) is similarly defined. The rank-based statistic *S*_*j*_ of marker *j* is then calculated to be the absolute value of the mean difference between {*R*_X*_(1, *j*),…, *R*_X*_(*n*_1_, *j*)} and {*R*_Y*_(1, *j*),…, *R*_Y*_(*n*_0_, *j*)}; that is,

(3) $$\begin{array}{c} S_{j}=\left|\frac{1}{n_{1}}\sum_{i=1}^{n_{1}}R_{X^{*}}\left(i,j\right)-\frac{1}{n_{0}}\sum_{i=1}^{n_{0}}R_{Y^{*}}\left(i,j\right)\right|. \end{array}$$

A relevant marker *j* then should possess a large value of *S*_*j*_. Note that the concept of “rank-over-variable” we adopt here to construct *R*_X*_(*i*, *j*) and *R*_Y*_(*i*, *j*) is more appropriate than the traditional rank-over-sample methods to analyze large-*p*-small-*n* datasets as described in Section 1.

**Figure 1:**
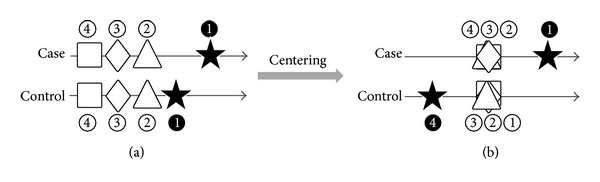
Rank-over-variable with or without centering. (a) Without centering, the rank of each marker in two groups has no difference. (b) With centering, the relevant marker has the highest rank in Case while it has the lowest rank in Control.

In the construction of (3), all the data points are subtracted by the overall mean *μ*_*j*_ before ranking over variables within a subject. We note that this *centering* is critical.  Figure 1 shows a simple example to illustrate its necessity. Consider four markers where one (with the symbol ⋆) is relevant and the remaining (with the symbols □, ⋄, △) are irrelevant to disease status. In control group, we assume that the mean of relevant marker (⋆) is larger than that of other three markers (Figure 1(a)). In case group, the means of irrelevant markers is identical to that in control group, while there is a large mean shift of marker ⋆. If we are only concerned about the order of the four markers in each group, we cannot observe any difference (Figure 1(a)). Obviously, rank-over-variable method without centering by means will fail to identify the relevant marker in this situation. Instead, supposing that all data points are subtracted by the overall means, the irrelevant markers will be close to zero while the relevant markers will be in the opposite directions and far away from zero as shown in Figure 1(b). In fact, the null hypothesis of the rank-over-variable method is that the interrelationships among the “centered markers” in case and control groups are the same; that is, 

*H*_0_: The orders of centered markers within two groups are identical. 

 Violation of the null hypothesis then indicates the existence of some relevant features, and those features can be reasonably identified by the ranking score (3).

If there are fewer irrelevant markers, the relevant markers might be more likely to be ranked in the top list. In most cases, however, the proportion of relevant markers is much lower than that of irrelevant markers, and the performance of the ranking list directly based on *S*_*j*_ may not be satisfactory. To enhance the detection power of the rank-based statistic *S*_*j*_, we further apply the techniques of “random subset” and “rerank” [9, 10] as described later. Here we use **I**(·) to denote an indicator function.

**Algorithm 1 (random subset)**

(1) Randomly select a marker index subset {*ℓ*_1_^(*b*)^,…, *ℓ*_⌈*p*/2⌉_^(*b*)^} of {1,…, *p*} without replacement to form the partial dataset with the selected ⌈*p*/2⌉ markers.

(2) Calculate {*S*_*j*_^(*b*)^ : *j* ∈ {*ℓ*_1_^(*b*)^,…, *ℓ*_⌈*p*/2⌉_^(*b*)^}} in (3) based on the dataset obtained in Step  1.

(3) Repeat Steps  1-2 for *b* = 1,…, *B*, and output the adjusted rank-based statistic

(4) $$\begin{array}{c} S_{j}^{*}=\frac{\sum_{b=1}^{B}S_{j}^{\left(b\right)}\cdot I\left(j\in \left\{\ell_{1}^{\left(b\right)},\ldots ,\ell_{\left\lceil p/2\right\rceil }^{\left(b\right)}\right\}\right)}{\sum_{b=1}^{B}\;I\left(j\in \left\{\ell_{1}^{\left(b\right)},\ldots ,\ell_{\left\lceil p/2\right\rceil }^{\left(b\right)}\right\}\right)}. \end{array}$$

The idea of random subset is intuitive: calculation of *S*_*j*_ with fewer irrelevant variables should be more efficient. Following the strategy of Chang and Chen [9], the size of subsets is chosen to be half of the number of markers in the original dataset. To take into account all combinations of markers and to ensure each marker is included with a sufficiently large number of times, the procedure is repeated *B* times.

The adjusted rank-based statistic *S*_j_*, however, is still calculated with the all markers being involved. Considering a ranking list of markers constructed by (4), we can reasonably regard the low-ranked markers as irrelevant markers. If we drop those irrelevant markers, the relevant markers might be more likely to be ranked in the top of the list. This fact motivates us to further consider the technique of “rerank,” and the algorithm is described later.

**Algorithm 2 (rerank)**

Initialize. *I*_0_ = {1,…, *p*} and *t* = 0

(1) Calculate {*S*_j_*^(*t*)^ : *j* ∈ *I*_*t*_} defined in (4) and

(5) $$\begin{array}{c} w_{t}=\frac{1}{p_{t}}\sum_{j\in I_{t}}\left(\frac{{S_{j}^{*}}^{\left(t\right)}}{p_{t}}\right), \end{array}$$

where *p*_*t*_ is the number of markers in *I*_*t*_.

(2) Based on {*S*_j_*^(*t*)^ : *j* ∈ *I*_*t*_}, collect the top *q*% markers in *I*_*t*_ to form *I*_*t*+1_.

(3) Repeat Steps  1-2 until *p*_*T*+1_ < *M*_1_, and output

(6) $$\begin{array}{c} S_{j}^{**}=\sum_{t=0}^{T}w_{t}\left(\frac{{S_{j}^{*}}^{\left(t\right)}}{p_{t}}\right). \end{array}$$

**Figure 2:**
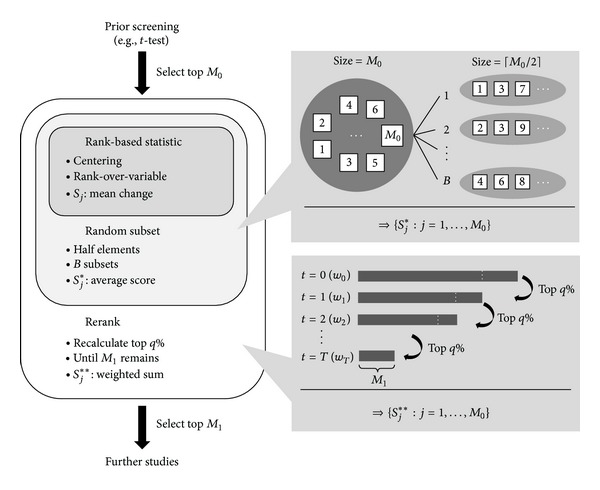
Flowchart of the rerank approach.

In the rerank procedure, we can reasonably expect that a relevant marker would be recalculated many times. It is straightforward to sum up all adjusted rank-based statistics *S*_j_*^(*t*)^ from each iteration, and then a relevant marker would possess a large score. However, there are two parts that should be modified. Firstly, note that the magnitude of rank-over-variable statistics will be influenced by the number of markers (*p*_*t*_) under consideration. To make statistics from different iteration comparable, we use *S*_j_*^(*t*)^/*p*_*t*_ instead of *S*_j_*^(*t*)^ in the rerank algorithm. Secondly, if the averaged score in an iteration is large, it implies that this iteration includes more markers with good separability of disease status. To implement this idea, we use the weight *w*_*t*_ defined in (5) to quantify the importance of each iteration. The final score *S*_j_** from rerank technique is therefore defined as a weighted sum in (6). Based on the ranking list constructed by {*S*_j_** : *j* = 1,…, *p*}, researchers can select *M*_1_ top-ranked markers as candidates for further evaluation. The flowchart of the proposed rerank approach is placed in Figure 2. In practice, the choice of *M*_1_ depends on research funding, prior knowledge, and so forth. A data-driven approach to determine *M*_1_ is developed in Section 2.2.

**Remark 3**

Both “random subset” and “rerank” are computationally demanded. To increase the computation speed, we suggest to select the top *M*_0_ markers by *t*-test, and the rerank approach is only implemented on these *M*_0_ markers to identify candidate features. It is verified in our simulation studies that this preprocessing does not affect the performance heavily.

### 2.2. Selection of *M*_1_

 Given a ranking list, researchers can select *M*_1_ top-ranked markers as candidates for further evaluation. For example, in our bipolar study in Section 3.2, it is allowed to select *M*_1_ = 100 due to the limited budget. In the case of having no prior knowledge about *M*_1_, *P* value and false discovery rate (*q*-value) are commonly used indices for feature selection. However, when the sample size is extremely small (e.g., 8 case and 8 control pools in our bipolar dataset), these methods may not be ready to be applied. In this study, we alternatively propose a method to directly estimate the number of truly relevant markers based on the constructed ranking list.

Consider a ranking list constructed by the rerank approach. If a marker is relevant, it implies that all of the higher-ranked markers in this ranking list are also relevant. Based on the idea of Cook and Yin [11], instead of computing a standard permutation-based *P* value for each marker, we compute a modified *P*-value *ν*_*m*_ to determine whether *all of the higher-ranked markers are relevant*. The algorithm is described later.

**Algorithm 4**

(1) Calculate {*S*_j_**^(0)^ : *j* = 1,…, *p*} defined in (6) and ${\overset{-}{S}}^{\left(0\right)}=(1/p)\sum_{j=1}^{p}{S_{j}^{**}}^{\left(0\right)}$.

(2) Construct a ranking list based on {*S*_j_**^(0)^ : *j* = 1,…, *p*}. Let *ℓ*_(*m*)_ be the index such that *S*_*ℓ*_(*m*)__^∗∗(0)^ is the *m*th largest.

(3) For *m* = 1,2,…, *p*,

(i) randomly permute the class labels on those markers with indices {*ℓ*_(*m*+1)_,…, *ℓ*_(*p*)_} to form a partially permuted dataset,
(ii) based on the dataset obtained from Step  3(i), calculate {*S*_j_**^(*b*)^:*j* = 1,…, *p*} defined in (6) and ${\overset{-}{S}}_{m}^{\left(b\right)}=(1/p)\sum_{j=1}^{p}{S_{j}^{**}}^{\left(b\right)}$,
(iii) repeat Step  3(i)-(ii) for *b* = 1,…, *B*, and output
  (7) $$\begin{array}{c} \nu_{m}=\frac{1}{B}\sum_{b=1}^{B}I\left({\overset{-}{S}}_{m}^{\left(b\right)}\geq {\overset{-}{S}}^{\left(0\right)}\right). \end{array}$$

We now describe the rationale of this algorithm and how to use the *ν*_*m*_ values to determine *M*_1_. Assume m* is the number of truly relevant markers and we are given a correct ranking list. Firstly, in the population level, it is obvious that *ν*_*m*_ is an increasing function of *m* provided the ranking list is correct. When *m* = m*, the algorithm actually permutes all irrelevant markers to form the permuted data, while the relevant markers are not permuted. In this case, the permuted data should behave very similarly to the original one and, hence, the distribution of ${\overset{-}{S}}_{m^{*}}^{\left(b\right)}$ should be identical to that of ${\overset{-}{S}}^{\left(0\right)}$. We thus expect that the value of *ν*_m*_ is close to 0.5. Moreover, when *m* > m*, *ν*_*m*_ is expected to be increasing uniformly in *m*, since we are including markers without separation abilities, and finally to reach unity when *m* = *p*. On the other hand, when *m* < m*, the relevant markers are permuted and the value of ${\overset{-}{S}}_{m}^{\left(b\right)}$ can be hardly as large as ${\overset{-}{S}}^{\left(0\right)}$. In this case, the *ν*_*m*_ value should be lower than 0.5, and the pattern of {*ν*_*m*_ : *m* < m*} should be far away from that of {*ν*_*m*_ : *m* > m*}. Based on the previous properties, we thus suggest to choose

(8) $$\begin{array}{c} M_{1}=\min\left\{m:\nu_{m}\geq 0.5\right\}. \end{array}$$

The proposed selection criterion will be evaluated by a simulation study as described later.

**Figure 3:**
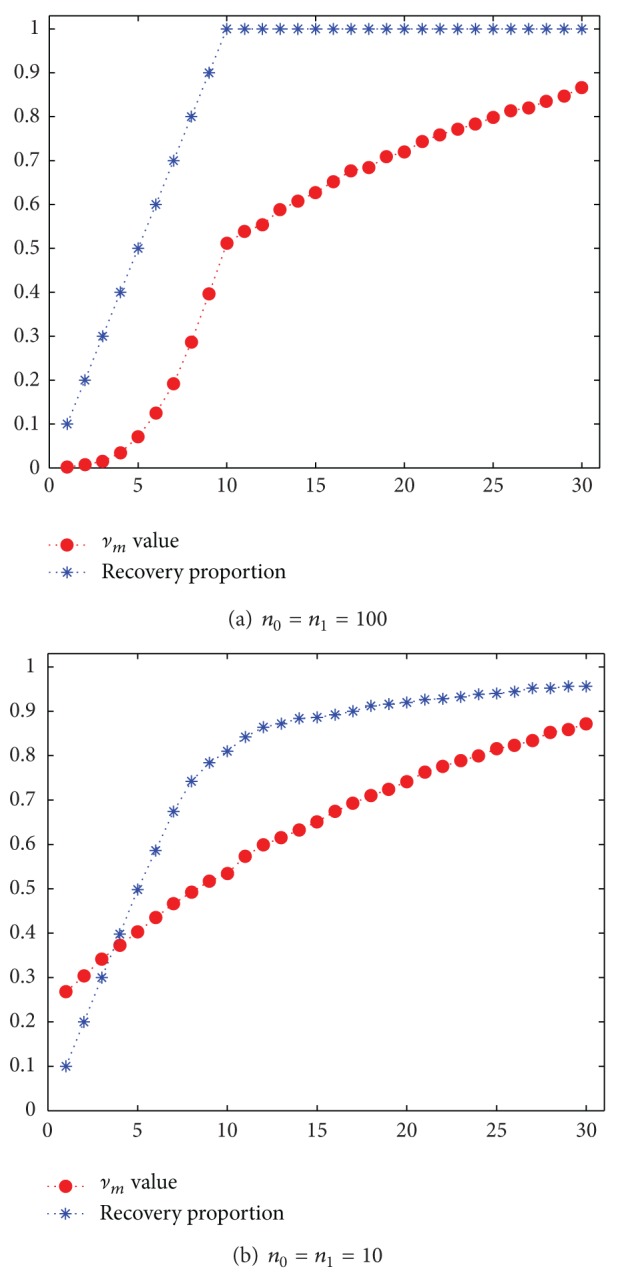
The average *ν*_*m*_ values and recovery proportion over 50 simulated datasets. The *x*-axis represents the number of unpermitted markers (*m*). Circles are the average *ν*_*m*_ values at *m*. Asterisk symbols are the average proportion of identified truly relevant markers included in the top *m* markers (recovery proportion).

We conduct a simulation study with 2000 markers for equal numbers of case and control groups. All of the markers in both groups follow standard normal distribution except m* = 10 markers in the case group that are distributed as *N*(2,1). With 100 cases and 100 controls, Figure 3(a) shows that the *ν*_*m*_ curve rises rapidly before reaching 0.5. After passing *ν*_*m*_ = 0.5, the curve increases uniformly with increasing *m*. An obvious change point at *m* = 10 with *ν*_10_ ≈ 0.5 suggests that *M*_1_ = 10 is a suitable choice. Figure 3(a) also shows that the recovery proportion is 100% when *m* = 10. When the sample size is merely 10 (Figure 3(b)), although there is no obvious change point, it still shows that the corresponding *m* of *ν*_*m*_ = 0.5 is close to m* = 10. The simulation results suggest that we can utilize the formula (8) to select a possible value of *M*_1_, after which the markers are treated as irrelevant.

## 3. Numerical Studies

The proposed rerank approach is applicable to analyze large-*p*-small-*n* datasets with continuous measurements. One application is the pooling-based genomewide association (GWA) study dataset. Instead of individual genotyping which is more expensive, using pooled DNA samples is an effective strategy to reduce the costs of GWA studies. In a pooling-based GWA study, the sample is genotyped in pools of individuals instead of individually genotyping. In particular, the data points *X*_*ij*_ (*Y*_*ij*_) from a pooling-based GWA study are the estimated allele frequencies for SNP *j* of pool *i* in the case (control) group. However, it will generate an ultrahigh dimensional dataset with extremely small sample size. For example, there are 249,473 markers but only 8 case and 8 control pools available in our bipolar dataset. Moreover, the additional measurement error from the pooling process and the existence of outliers have the potential to decrease detection power. In this situation, the proposed rerank approach is more suitable to deal with the large-*p*-small-*n* datasets and is more robust to the pooling error and outliers. These facts will be confirmed by the following numerical studies.

### 3.1. Simulation Studies Using GAIN-MDD Dataset

We simulate DNA pooling datasets from a real individual genotype dataset called GAIN-MDD dataset, which was accessed through the Genetic Association Information Network (GAIN) studies database of Genotypes and Phenotypes (dbGaP) for major depressive disorder (MDD) [12, 13]. There are 416,170 SNPs with 1673 cases and 1721 controls after quality control. We first implement the basic case/control association test by PLINK [14] to the original GAIN-MDD dataset and then define the top 100 SNPs as the truly relevant SNPs. To simulate a pooling-based GWA dataset, *n*_1_ case pools and *n*_0_ control pools are constructed by randomly selecting *n*_1_ × *s* cases and *n*_0_ × *s* controls from GAIN-MDD dataset, where *s* is the pooling size. Let ${\tilde{X}}_{ij}$ and ${\tilde{Y}}_{ij}$ be the minor allele frequency (MAF) for SNP *j* of pool *i* in the case and control groups, respectively. To mimic the existence of pooling error and outliers, the observed MAF is generated by

(9) $$\begin{array}{c} X_{ij}=\frac{\exp\left(\log({\tilde{X}}_{ij}/\left(1-{\tilde{X}}_{ij}\right))+\varepsilon \right)}{1+\exp\left(\log({\tilde{X}}_{ij}/\left(1-{\tilde{X}}_{ij}\right))+\varepsilon \right)},\;\varepsilon \sim N\left(0,\sigma^{2}\right), \end{array}$$

and *Y*_*ij*_ is similarly defined. Collect {*X*_*ij*_ : *j* = 1,…, 416170}_*i*=1_^*n*_1_^ and {*Y*_*ij*_ : *j* = 1,…, 416170}_*i*=1_^*n*_0_^ to form a simulated DNA pooling dataset. The anticipated aim of this simulation study is to recover the 100 truly relevant SNPs by analyzing the simulated DNA pooling dataset. We repeat simulation studies 100 times and report the averaged number of truly relevant SNPs identified in the top ranking list of each method. We use *M*_0_ = 5000, *B* = 100, and *q* = 87.5 for rerank approach. The SAM is implemented by the *samr* R package (from http://www-stat.stanford.edu/~tibs/SAM/) [6]. As to the setting of *σ*, there are three situations considered: (A) without pooling error and outliers (*σ* = 0); (B) with pooling error (*σ* = 0.05); and (C) 1% data points are outliers with *σ* = 5, and *σ* = 0.05 otherwise.

**Figure 4:**
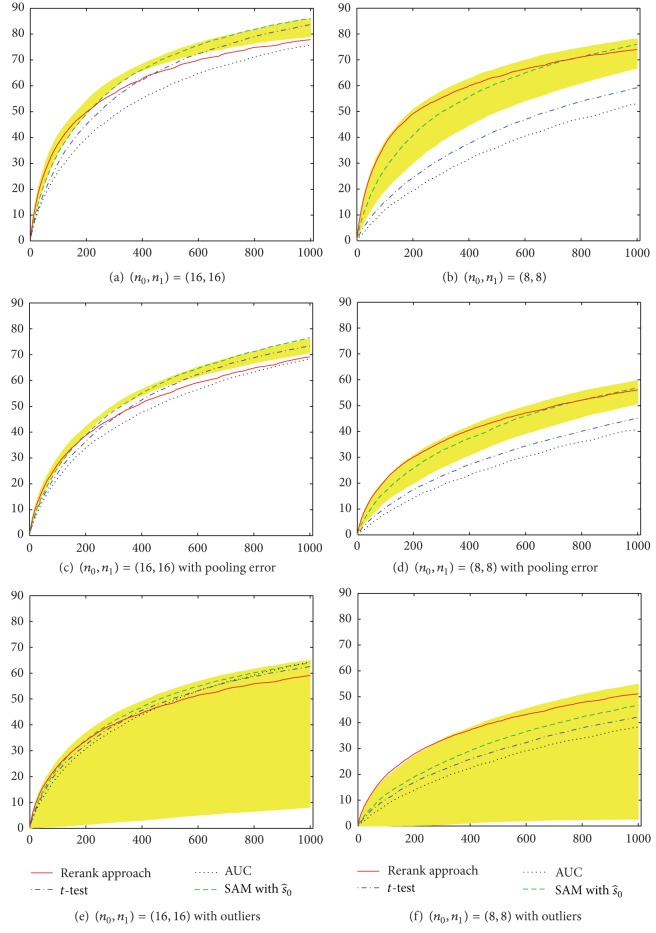
Performance comparison of different methods for feature selection with equal sample size in two groups. The *x*-axis represents the number of selected SNPs (*M*_1_) based on simulated DNA pooling data. The *y*-axis represents the average number of identified truly relevant SNPs over 100 simulations. The shaded area represents the possible results of SAM with various *s*_0_. The ${\hat{s}}_{0}$ value is automatically determined by *samr* R package.

**Figure 5:**
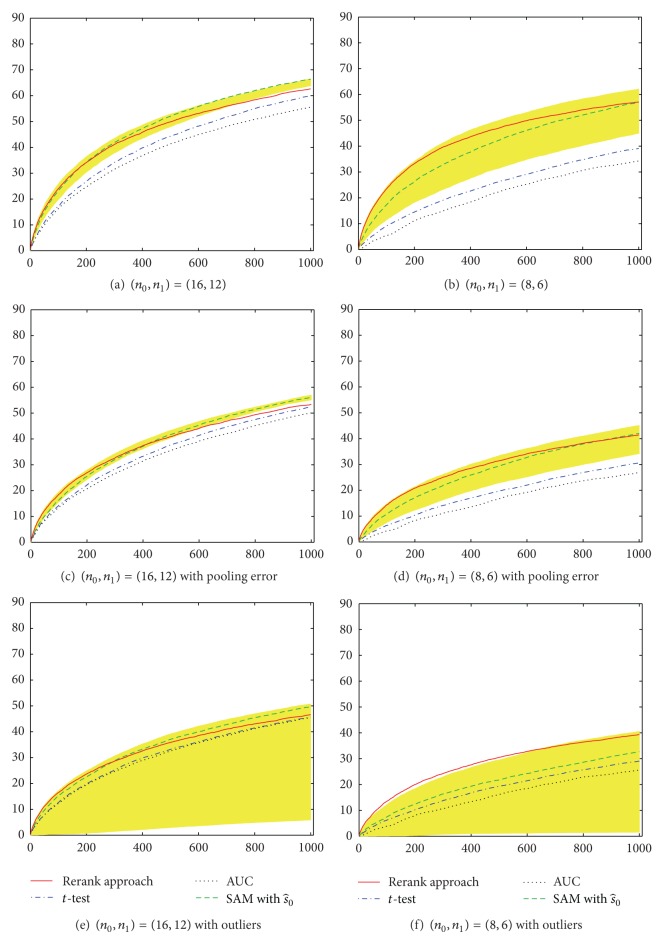
Performance comparison of different methods for feature selection with unequal sample size in two groups. The *x*-axis represents the number of selected SNPs (*M*_1_) based on simulated DNA pooling data. The *y*-axis represents the average number of identified truly relevant SNPs over 100 simulations. The shaded area represents the possible results of SAM with various *s*_0_. The ${\hat{s}}_{0}$ value is automatically determined by *samr* R package.

Simulation results for equal numbers of case and control pools are shown in Figure 4. When *σ* = 0, under the case of *n*_0_ = *n*_1_ = 16 (each with size *s* = 100), Figure 4(a) shows that the performance of rerank approach is better than AUC for any *M*_1_ and is better than *t*-test except for large *M*_1_. Note that the 100 truly relevant SNPs are selected based on chi-square test, which is similar to *t*-test, and it is reasonable for *t*-test to obtain similar ranking list of susceptible SNPs, even when data was aggregated. For the case of *n*_0_ = *n*_1_ = 8 (each with size *s* = 200), Figure 4(b) shows that *t*-test and AUC have worse performances due to the instability in estimating variances and high proportion of tied values, respectively. In contrast, the proposed rerank approach does outperform these two methods for any given *M*_1_. In the presence of pooling errors (Figures 4(c) and 4(d)) and outliers (Figures 4(e) and 4(f)), the performances of all methods become worse, but a similar pattern can be observed. The similar patterns can be also observed when the numbers of case and control pools are unequal, except that the performances of all methods become worse simultaneously (Figure 5).

In Figures 4 and 5, we also plot the results of SAM with various choices of *s*_0_, and with the estimated ${\hat{s}}_{0}$ by *samr* R package [6]. Observing the shaded area from SAM with various *s*_0_, SAM has a chance to identify more truly relevant SNPs than other methods, provided that we can accurately choose the optimal *s*_0_* (which corresponds to the highest line of the shaded area). Unfortunately, *s*_0_* is unknown in advance and there is no guarantee that the suggested algorithm by [6] can choose ${\hat{s}}_{0}=s_{0}^{*}$. See the dashed line in Figures 4 and 5, which is far from the optimal result of SAM. Moreover, the wide range of shaded areas indicates that the choice of *s*_0_ is critical to the performance of SAM, especially for small sample size and in the presence of outliers. On the other hand, the performance of the rerank approach is similar to that of SAM with optimal *s*_0_* for small *M*_1_ and is better than SAM with the estimated ${\hat{s}}_{0}$ for a wide range of *M*_1_.

**Figure 6:**
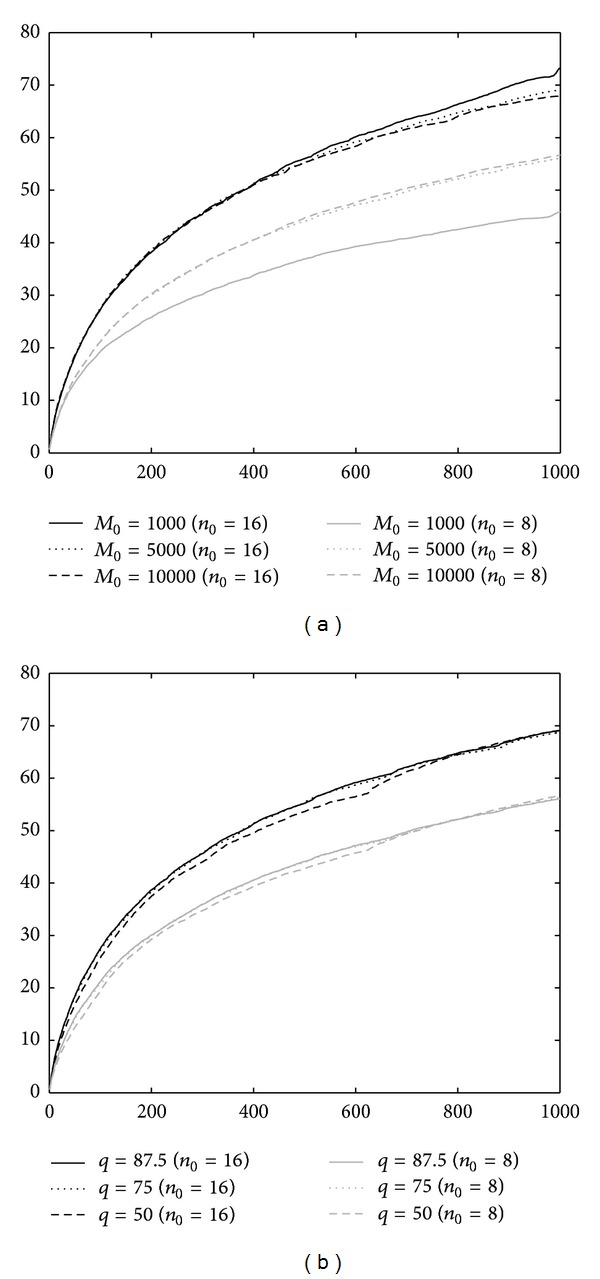
Performance comparison under different *M*_0_ and *q* for rerank approach under the case of *n*_0_ = *n*_1_ and *σ* = 0.05. The *x*-axis represents the number of selected SNPs (*M*_1_) based on simulated DNA pooling data. The *y*-axis represents the average number of identified truly relevant SNPs over 100 simulations.

Another advantage of our rerank approach is its insensitivity to the selection of tuning parameters. To see this, we further report the simulation results for various *M*_0_ (the number of prescreened SNPs) and *q*% (the percentage of rerank) values under equal numbers of case and control pools and *σ* = 0.05.  Figure 6(a) suggests that we should choose a conservative value of *M*_0_ (e.g., larger than 5000) especially for small sample size (*n*_0_ = *n*_1_ = 8), although there is no obvious difference for larger sample size (*n*_0_ = *n*_1_ = 16).  Figure 6(b) shows that the performance is not sensitive to *q* except for the case of *q* = 50. These simulation results then suggest to use a conservative value of *M*_0_ and *q*, and the performance of rerank approach is guaranteed. In summary, the rerank approach is more robust to small sample size, pooling error, and outliers and is insensitive to the selection of tuning parameters.

### 3.2. Bipolar Dataset

**Figure 7:**
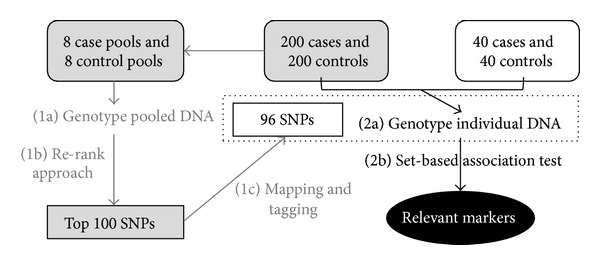
Flow diagram of DNA pooling-based two-stage GWA study design. Stage 1 is denoted by grey color, and Stage 2 is denoted by black color.

In this subsection, we demonstrate a real data analysis using the proposed rerank approach. The dataset is from a two-stage GWA study to identify common variants for the association with bipolar disorder [15]. The bipolar disorder patients were recruited from three hospitals in southern Taiwan from 2008 to 2010. Healthy controls were recruited from the community through advertisements. At Stage 1, a genomewide screen using Illumina HumanOmini1-Quad chip with 970,342 SNPs was performed by DNA pooling with 8 case and 8 control pools constructed from 200 patients and 200 controls. Among the initial 970,342 SNPs, we exclude SNPs if they are (1) on sex chromosome, (2) failed genotyping, (3) monomorphic, (4) with call rate <0.8, or (5) with MAF <0.05. After quality control filtering, there remain 249,473 SNPs. The rerank approach is then applied to evaluate the association for each SNP and to construct a ranking list. One hundred top-ranked SNPs are selected to design and make a panel with 96 SNPs, which would be individually genotyped in Stage 2 with the original plus additional samples, with the aim of identifying relevant SNPs responsible for bipolar disorder. We also aim to see if the result based on pooling data can be reproduced by individual genotype data, to evaluate the performance of DNA pooling for SNP selection. The flow diagram of this analysis is shown in Figure 7.

**Figure 8:**
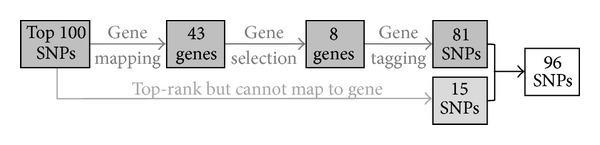
Selection process for the 96 SNPs in Stage 2 of Bipolar study.

Recall the aim of Stage 1 is to design a panel with 96 SNPs for that validation by individually genotyping in Stage 2. The selection process is shown in Figure 8. We first select top 100 SNPs by the rerank approach with *M*_1_ = 100, *B* = 100 and *q* = 87.5. Among those SNPs, 52 SNPs do not map to any gene while the remaining SNPs can map to 43 genes totally. According to previous studies, etiology of bipolar disorder involves neurotransmitter, neuronal system, immune function, and brain development. Among the 43 genes, we only focus on 8 genes that are associated with brain or neuron. They can be categorized into different biological functions, such as brain-specific chemokines or neurokines and receptor or ligand that regulates neuronal positioning or axon guidance. We next select 81 tag SNPs for the 8 genes based on Tagger [16]. In addition, we choose 15 SNPs that are top-ranked but cannot map to any gene. The total 96 SNPs are conducted in a panel for Stage 2. Using the individual genotype data from Stage 2, the association test for each SNP is implemented in PLINK by fitting simple logistic regression under allelic, dominant, recessive, and additive genetic models, respectively [14].

To evaluate the reproducibility of the findings from Stage 1, Table 1 shows the association analysis results for the 16 overlapping SNPs in both Stage 1 and Stage 2, where the odds ratio (OR) and *P*-value in Stage 2 are based on the genetic model with the most significant result. Among the 16 SNPs, 13 of them attain 5% significant level in Stage 2 wherein 6 markers are positively relevant to bipolar disorder (OR = 1.4*∼*1.5) and 7 markers are negatively relevant (OR = 0.5*∼*0.7). One can see that the analysis results from Stage 1 and Stage 2 are consistent. It implies that the susceptible markers identified by our rerank approach have high reproducibility even using the pooled DNA data. Those relevant markers (*P*-value < 0.05), however, are listed in the much lower rank of *t*-test. The poor performance of *t*-test can be improved by SAM, but it still could not perform as efficient as the rerank approach did. For example, the SNP 3, SNP 6, and SNP 8 have very small *P*-values (0.00085, 0.00651, 0.00785), but not in the top list of SAM.

To validate the findings from Stage 1, we further conduct a set-based analysis for the 8 selected genes under different genetic models of association test by PLINK [14] using individual genotype data from the original plus additional samples.  Table 2 shows that the Gene 5 attains 5% significance level under all models of set-based analysis from Stage 2. Under some genetic models, the Gene 4, Gene 6, Gene 7, and Gene 8 are also significant.

Based on the previously mentioned replication and validation steps by analyzing individual genotype data, our rerank approach indeed has the ability to efficiently detect associations using pooled DNA data. Researchers can focus on these candidate SNPs or genes for further biological studies.

## 4. Conclusions

 In this study, we propose a robust rerank approach to create a ranking list for feature selection, which comprises three components: (1) rank-based statistic (rank-over-variable), (2) random subset, and (3) rerank. The rank-based statistic is the main scoring function for quantifying association strength, which is motivated by the Rank Test of Alvo et al. [8]. We also apply the techniques of random subset and rerank [9, 10] iteratively to enhance the detection power of rank-based statistic. The combination of these three components demonstrates good performance and robustness in both simulation and real pooling-based GWA study datasets. In addition to the pooling-based GWA study datasets, our rerank approach can be applied to any large-*p*-small-*n* datasets with continuous measurements to select differential features between two groups, such as gene expression datasets and biomarker datasets. It provides researchers a sizeable number of differential features for further studies.

In the rerank approach, it involves an important concept: rank-over-variable. The advantage is not only to avoid tied values for ranking in the large-*p*-small-*n* situation, but the information of correlations among features can also be taken into account during the ranking process. In other words, although the rank-based statistic is defined as the mean difference of rank values, it is likely to have the ability to detect both mean and correlation changes between two groups [8]. It is interesting to investigate this mechanism in a future study.

## Figures and Tables

**Table 1:** Association analysis of the 16 overlapping SNPs in both stages of bipolar study.

SNP	Chr	Pooling (Stage 1)					Individual genotyping (Stage 2)				
		RowSpanEmpty	*R*	*T*	SAM	CA	CN	CA	CN	Model	OR	*P* value
SNP 1	3	7	189	5	0.17	0.36	0.21	0.35	DOM	0.5	0.00002*
SNP 2	3	2	363	15	0.23	0.43	0.22	0.33	DOM	0.6	0.00018*
SNP 3	6	28	873	152	0.53	0.38	0.45	0.33	ALL	1.6	0.00085*
SNP 4	4	36	124	58	0.31	0.44	0.33	0.44	ALL	0.7	0.00142*
SNP 5	21	13	531	64	0.58	0.41	0.55	0.43	ADD	1.5	0.00294*
SNP 6	6	41	1308	346	0.41	0.28	0.34	0.25	ADD	1.5	0.00651*
SNP 7	16	10	110	18	0.33	0.49	0.34	0.45	ALL	0.7	0.00725*
SNP 8	13	48	596	126	0.56	0.42	0.52	0.40	ALL	1.4	0.00785*
SNP 9	2	101	7484	2795	0.50	0.40	0.48	0.41	ADD	1.4	0.00960*
SNP 10	4	5	19	1	0.27	0.46	0.27	0.36	ALL	0.7	0.00971*
SNP 11	14	40	4678	1025	0.36	0.49	0.42	0.53	DOM	0.7	0.01275*
SNP 12	18	20	393	47	0.49	0.33	0.47	0.36	ALL	1.4	0.01604*
SNP 13	3	6	694	44	0.33	0.53	0.39	0.49	DOM	0.7	0.01631*
SNP 14	7	8	36	2	0.31	0.47	0.37	0.45	ADD	0.8	0.06830
SNP 15	5	21	762	57	0.37	0.56	0.35	0.42	DOM	0.8	0.09806
SNP 16	7	22	210	21	0.35	0.53	0.31	0.38	ALL	0.8	0.13530

Chr: chromosome; *R*: rank based on rerank approach; *T*: rank based on *t*-test; SAM: rank based on SAM with ${\hat{s}}_{0}$; CA: MAF of cases; CN: MAF of controls; Model: genetic model with the most significant result among allelic (ALL), dominant (DOM), recessive (REC), and additive (ADD) genetic models; OR (odds ratio) and P value: estimated by the simple logistic regression under the genetic model with the most significant result (*P value < 0.05).

**Table 2:** Set-based analysis of the 8 selected genes under different models of association test.

Gene	NSNP	Allelic model			Dominant model			Recessive model			Additive model		
		RowSpanEmpty	NSIG	ISIG	EMP	NSIG	ISIG	EMP	NSIG	ISIG	EMP	NSIG	ISIG	EMP
Gene 1	11	0	0	1.000	1	1	0.375	0	0	1.000	0	0	1.000
Gene 2	12	1	1	0.443	1	1	0.069	0	0	1.000	1	1	0.415
Gene 3	10	0	0	1.000	0	0	1.000	0	0	1.000	0	0	1.000
Gene 4	9	2	1	0.057	2	1	0.190	1	1	0.135	2	1	0.005*
Gene 5	11	4	2	0.008*	3	2	0.011*	1	1	0.008*	4	2	0.005*
Gene 6	10	1	1	0.007*	2	2	0.129	1	1	0.054	1	1	0.010*
Gene 7	12	1	1	0.107	1	1	0.038*	0	0	1.000	1	1	0.088
Gene 8	4	1	1	0.042*	0	0	1.000	1	1	0.046*	1	1	0.037*

NSNP: number of SNPs in a set; NSIG: total number of SNPs below *P* value with 0.05; ISIG: number of significant SNPs also passing LD criterion on an *r*-squared threshold of 0.5; EMP: empirical set-based *P* value (*EMP < 0.05).
